# Plasticity of Human CD4 T Cell Subsets

**DOI:** 10.3389/fimmu.2014.00630

**Published:** 2014-12-16

**Authors:** Jens Geginat, Moira Paroni, Stefano Maglie, Johanna Sophie Alfen, Ilko Kastirr, Paola Gruarin, Marco De Simone, Massimiliano Pagani, Sergio Abrignani

**Affiliations:** ^1^Fondazione Istituto Nazionale di Genetica Molecolare “Romeo ed Enrica Invernizzi” INGM, Milan, Italy

**Keywords:** CD4 T cells, cytokines, differentiation, tissue homing, plasticity

## Abstract

Human beings are exposed to a variety of different pathogens, which induce tailored immune responses and consequently generate highly diverse populations of pathogen-specific T cells. CD4^+^ T cells have a central role in adaptive immunity, since they provide essential help for both cytotoxic T cell- and antibody-mediated responses. In addition, CD4^+^ regulatory T cells are required to maintain self-tolerance and to inhibit immune responses that could damage the host. Initially, two subsets of CD4^+^ helper T cells were identified that secrete characteristic effector cytokines and mediate responses against different types of pathogens, i.e., IFN-γ secreting Th1 cells that fight intracellular pathogens, and IL-4 producing Th2 cells that target extracellular parasites. It is now well established that this dichotomy is insufficient to describe the complexity of CD4^+^ T cell differentiation, and in particular the human CD4 compartment contains a myriad of T cell subsets with characteristic capacities to produce cytokines and to home to involved tissues. Moreover, it has become increasingly clear that these T cell subsets are not all terminally differentiated cells, but that the majority is plastic and that in particular central memory T cells can acquire different properties and functions in secondary immune responses. In addition, there is compelling evidence that helper T cells can acquire regulatory functions upon chronic stimulation in inflamed tissues. The plasticity of antigen-experienced human T cell subsets is highly relevant for translational medicine, since it opens new perspectives for immune-modulatory therapies for chronic infections, autoimmune diseases, and cancer.

## Introduction

Human CD4^+^ T cells are critical regulators of the immune system, as drastically demonstrated by HIV-infected individuals that develop susceptibility to opportunistic infections and cancer when virus-dependent depletion reduces CD4^+^ T cell counts below critical thresholds (1). CD4^+^ T cells are very heterogeneous in human adults, because they have been generated in response to a high number of different pathogens and belong to a progressively increasing number of different subsets with specialized functions (2). Helper T cell subsets are defined by the production of cytokines and/or the expression of characteristic lineage-defining transcription factors (Table 1). Five principal subsets or lineages of CD4^+^ T cells have been identified so far: T helper (Th)1, Th2, and Th17 cells that target specific classes of pathogens (3–5), regulatory T cells that are required to maintain self-tolerance (6) and follicular helper T cells (T_FH_) that provide help to B cells for antibody production (7). Heterogeneity is generated upon T cell priming, since naïve T cells have stem-cell-like properties and can differentiate into virtually all different types of effector, memory, or regulatory cells (Table 1). Antigen-experienced T cells are less flexible, but many subsets retain some plasticity and can acquire additional cytokine producing capacities upon antigenic re-stimulation, while others appear to be terminally differentiated (8). In some cases, T cell functions can even completely change from helper to regulatory functions (9) or vice versa (10). A caveat of these findings in particular in humans is the enormous heterogeneity of T cells (2), making it difficult to exclude a selective outgrowth of rare pre-existing precursor cells. Several excellent reviews on the plasticity of mouse T cells have been published in recent years (11–13), while human T cell plasticity is less understood, but highly relevant for new therapeutic strategies in immune-mediated diseases (14).

**Table 1:** **Phenotype, characteristics and functions of relevant human T cell subsets**.

T cell subset	Phenotype	Characteristic cytokines	Characteristic transcription factors	Function
Naïve	CD45RA^+^CCR7^+^	IL-2		Precursor cells, protection against new pathogens
T_CM_ (central memory)	CD45RA^−^CCR7^+^	IL-2, IL-21		Secondary expansions, help
T_EM_ (effector memory)	CCR7^−^	IFN-γ, IL-4, IL-5, IL-17		Protection in tissues, help
T_RM_ (tissue-resident memory)	CD103^+^CD69^+^	IFN-γ		Immediate protection in tissues
T_FH_ (follicular helper)	CXCR5^+^ICOS^+^	IL-21	BCL6	B cell help
Th1	CXCR3^+^	IFN-γ	T-bet	Protection against intracellular pathogens
Th2	CRTH2^+^	IL-4, IL-5, IL-13	GATA-3	Protection against extracellular parasites
Th9	?	IL-9	PU.1	Protection against extracellular parasites
Th17	CCR6^+^CD161^+^	IL-17, IL-22, IL-26	RORC2	Protection against extracellular bacteria and fungi
Treg	CD25^+^CD127^−^	TGF-β	FOXP3	Maintenance of self-tolerance
Tr1 (type 1 regulatory)	CD25^−^CD127^−^or CD49b^+^LAG3^+^	IL-10	?	Inhibition of immunopathology

## Terminally Differentiated TH1 and TH2 Effector Cells: The Tip of the Iceberg

Seminal studies have established that CD4^+^ T cells can differentiate into two types of effector cells with different cytokine producing capacities and functions in humans and mice (3, 4). Uncommitted naïve T cells that are activated by specialized dendritic cells that produce IL-12 (15, 16) acquire IFN-γ producing capacities. These so-called T helper 1 cells (Th1) are induced upon infections with intracellular pathogens like bacteria or viruses and can activate macrophages to destroy intracellular bacteria. In contrast, naïve T cells primed in the presence of IL-4 undergo a different fate and start to produce IL-4, IL-5, IL-10, and IL-13, but not IFN-γ. These Th2 cells are required to fight extracellular parasites like helminths, but since they induce IgE from B cells they are also involved in allergies (17). Importantly, it was shown that Th1 versus Th2 differentiation was a crucial decision to resist infections, since BL/6 mice that mount a Th1 response to leishmania were protected, while BALB/c mice that instead induce a Th2 response were highly susceptible (18). The characteristic cytokines produced by Th1 and Th2 cells, IFN-γ, and IL-4, were further shown to inhibit the differentiation to the opposite differentiation lineage and thus reinforced the original fate decision. The capacity to produce either IFN-γ or IL-4 is stably imprinted by epigenetic modifications like DNA methylation and histone acetylations, ensuring that the cytokine profile of T helper cells is preserved upon cellular division independently of the inducing polarizing cues (19–21). Moreover, the generation of Th1 and Th2 cells was shown to depend on the “master” transcription factors T-bet and GATA-3, which induced not only the characteristic cytokines of Th1 and Th2 cells, but also inhibited the differentiation to the alternative lineage. Based on this evidence, it was initially assumed that the differentiation to Th1 and Th2 cells are mutually exclusive and irreversible fate decisions.

## TH1 and TH2 Cells Can Acquire New Properties and Functions in Secondary or Chronic Immune Responses

Early studies with human T cell clones showed that IFN-γ and IL-4 production were not necessarily two exclusive features, since some T cells co-produced IFN-γ and IL-4 (22). Notably, human Th1 memory cells are responsive to IL-4 stimulation, and acquire IL-4 producing capacities upon TCR stimulation in the presence of IL-4 without losing IFN-γ production *in vitro* (23).

In addition, some T cells in human blood co-express the Th1 and Th2 markers CXCR3 and CCR4 (24) or CRTh2 as well as the lineage-defining transcription factors GATA-3 and T-bet (25). Consistently, it was shown in mice that histones of these transcription factor genes had both repressive and permissive marks in opposing T cell lineages (13, 26). In mice, *in vivo* primed Th2 cells can acquire IFN-γ producing capacities in addition to IL-4 in response to IFN and IL-12 (27), while human blood Th2 cells seem to be less plastic (23). Moreover, the pathogens and the physiological conditions that induce Th1/2 cells in humans and their role in immune responses remain to be fully defined (25).

**Figure 1:**
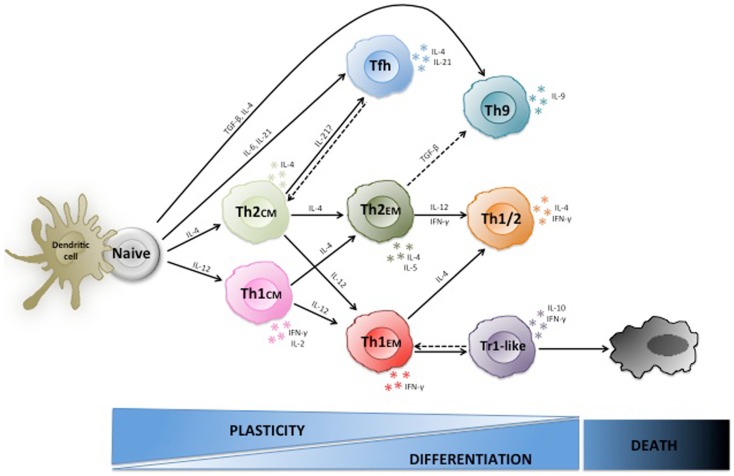
**Plasticity of human Th1 and Th2 cells**. Naive CD4^+^ T cells are stem-cell-like cells that under the influence of different cytokines can differentiate to various types of effector cells including Th1, Th2, Th9, and T_FH_ cells. Th1 and Th2 central memory cells are arrested at an early stage of differentiation, are highly plastic and some can still switch lineage. Conversely, effector memory cells are more differentiated, less plastic, and rather become polyfunctional. Moreover, Th1 effector cells can acquire IL-10 producing capacities and regulatory functions in chronically inflammed tissues.

Another early finding that did not fit well into the fixed Th1/Th2 paradigma was the fact that IL-12 could induce IL-10 in Th1 cell clones (28). IL-10 has potent anti-inflammatory functions and inhibits maturation and T cell stimulatory capacities of APC (29), thus the concomitant expression of both IFN-γ and IL-10 by T cells was unexpected (30). Later it was shown that IL-10 produced by T-bet^+^ Th1 cells was required to inhibit lethal immunopathology upon infections with intracellular parasites (31, 32), indicating that IL-10-producing Th1 cells prevent overshooting immune responses and the resulting tissue damage in a negative feedback loop (9). Interestingly, although these IL-10 producing Th1 cells inhibited IL-12 production by APC, they were also able to restrict parasite growth via IFN-γ (31). However, IFN-γ has also been shown to have some negative effects on T cell responses (33, 34), providing a possible alternative explanation for IFN-γ production by regulatory T cells. Importantly, IFN-γ/IL-10 co-producing T cells with regulatory functions are present at low frequencies in peripheral blood of healthy donors and respond selectively to persistent pathogens (35), suggesting that similar to their mouse counterparts they inhibit overshooting immune responses in chronic infections. Thus, Th1 cells can switch from pro-inflammatory effector cells to IL-10 producing type 1 regulatory (Tr1)-like T cells (36, 37), and this switch is necessary to maintain the integrity of infected tissues in some infections. Complement receptor stimulation (38), production of IL-27 (39) or IL-12 (28) by myeloid cells (40), or generation of AHR ligands (41) are possible inductive cues, but also chronic or repetitive antigenic stimulation seems to be required to induce IL-10 production in Th1 cells (35, 42, 43). Interestingly, a recent paper suggests that IL-10/IFN-γ co-producing T cells can also be generated from Th17 cells under the influence of IL-12 or IL-27 in mice (44). If IFN-γ/IL-10 co-producing regulatory T cells are stably maintained or are short-lived, if they progressively lose IFN-γ production upon chronic stimulation or revert to Th1 cells upon pathogen clearance is currently unclear (Figure 1).

More recently, additional plasticity of Th2 cells was documented. Thus it was shown that T_FH_ cells were derived from Th2 precursor cells in mouse models of helminth infections (45). This finding is relevant for Th2 stability, because T_FH_ cells are professional B helper T cells that secrete IL-21 in B cell follicles, express the transcriptional repressor BCL-6 and are thus distinct from conventional Th1 and Th2 cells (7, 46, 47). Also in human tonsils a fraction of T_FH_ cells express the Th2 marker CRTH2 and produce IL-4 (48). The relationship of Th1 cells with T_FH_ cells is less clear in particular in humans (49, 50). Some murine T_FH_ cells produce IFN-γ (51), which induces IgG2a production by B cells (52), but T_FH_ cells from human tonsils lack IFN-γ production.

Mouse Th2 cells can also switch from IL-4 to IL-9 production upon stimulation with TGF-β (53). These Th9 cells express the PU.1 transcription factor (54) and can also be directly induced from naïve and memory T cells upon stimulation with TGF-β and IL-4 in humans and mice (55, 56). Th9 cells can have a pro-inflammatory role in allergic asthma (57) and respond to helminth antigens and allergens in humans (58, 59). However, IL-9 induction by TGF-β is not restricted to Th2 cells (60).

Collectively, these findings indicate that both Th1 and Th2 cells can acquire different cytokine producing capacities and functional properties upon antigenic re-stimulation under the influence of cytokines, and are thus much more flexible than originally thought (Figure 1).

## Stability of FOXP3^+^ Tregs is Debated

CD25^+^ regulatory T cells are required to maintain self-tolerance. They were first identified in mice (61) and later in humans (62), and the Foxp3 transcription factor was shown to be required for their generation and function (63, 64). Consistently, IPEX patients, who suffer from a devastating autoimmune disease, were found to have mutations in the Foxp3 gene (65). Although so-called natural or thymic Foxp3^+^ Tregs acquire regulatory lineage commitment already upon maturation in the thymus (66), adaptive, or peripheral Foxp3^+^ Tregs can be induced from mature CD4^+^ helper T cells in the periphery under the influence of TGF-β (67, 68). The transcription factor Helios was proposed to distinguish between these two subsets of natural and induced Foxp3^+^ Treg, but this concept was not confirmed by others (69–71). In humans, CD45RA^+^CD25^+^Foxp3^+^ cells represent a population of *bona fide* “naïve” and thus thymus-derived Tregs, while CD45RA^−^Tregs are a mixed population that contain antigen-experienced Tregs of both thymic and peripheral origin (72). The stability of Foxp3^+^ Tregs is debated (73). Lineage tracing of Foxp3^+^ T cells in mice has lead to conflicting interpretations, since in several studies only very small fractions of Foxp3^+^ Tregs were found to lose Foxp3 and regulatory functions *in vivo* (74). In humans, CD45RA^+^ but not CD45RA^−^ Tregs could be stably expanded *in vitro* (72, 75), suggesting different stabilities of thymic and peripheric Tregs. However, since human Tregs have to be purified according to surface marker expression, it is difficult to exclude a selective outgrowth of Foxp3^−^ cells or of activated effector T cells that have transiently up-regulated Foxp3 upon stimulation (73).

The functional specialization of Foxp3^+^ Treg is shaped by the tissue microenvironment (76), and the induction of transcription factors characteristic for helper T cell lineages in mice allows Tregs to suppress the corresponding T helper cell responses (74). Thus, STAT3 in Tregs is required to suppress Th17 cells (77), IRF4 to control Th2 responses (78) while Tregs that regulate T_FH_ cells and antibody responses express BCL-6 (79, 80). Foxp3^+^ Tregs also acquire T-bet and IFN-γ producing capacities upon stimulation with IL-12, and these Th1regs might be specialized to suppress Th1 responses (14, 74). Tregs also inhibit anti-tumor CTL responses (81), and interestingly they can acquire cytotoxic properties in tumor-draining lymph nodes in mice (82) and *in vitro* in humans (83), and tumor-infiltrating Tregs are consequently cytotoxic (84). Similar to helper T cells, Tregs that secrete different types of effector cytokines can be identified according to chemokine receptor expression (2), and these Treg subsets might specifically suppress different types of immune responses (85). Human Foxp3^+^ T cells that produce IL-17 or IFN-γ can be isolated (86, 87), but while IL-17 producing Treg cells were normally suppressive (88), IFN-γ producing Tregs had reduced suppressive functions (87). The conditions that induce human Foxp3^+^ Tregs to secrete different effector cytokines and the role of these cells in infections, cancer, and autoimmune diseases remain to be fully established.

## Heterogeneity and Unstability of TH17 Cells and Its Relevance for Autoimmune Diseases

The discovery of IL-17 producing helper T cells (Th17) in mice (89, 90) and humans (91) and their relative instability (11, 92) has led to a profound re-evaluation of the concept of two terminally differentiated helper T cell subsets. The fact that human CD4^+^ T cells produce IL-17 was known for a long time (93). However, it took a decade to realize that these cells represented an independent differentiation lineage (89, 90), which have unique differentiation requirements and express the lineage-defining transcription factor ROR-γt in mice and RORC2 in humans (94, 95). Th17 cells are important to fight extracellular bacteria and fungi, since patients that lack Th17 cells have uncontrolled infections with *Candida albicans* (*C. albicans*) and *Staphylococcus aureus* (96). The discovery of Th17 cells has been complicated by the fact that T cell differentiation to Th1 and Th17 cells relies on shared components of cytokines and their receptors. Thus, it was known that IL-12p40 and IL-12Rβ1 hetero-dimerize with respectively IL-12p35 and IL-12Rβ2 to induce Th1 cells, but later it was realized that they can also associate with respectively IL-23p19 and the IL-23R to promote Th17 responses (97). The IL-23/IL-23R pathway is involved in many different autoimmune diseases (98–100) and IL-23-induced Th17 cells are thought play a prominent pathogenic role (101–104). Conversely, the contribution of Th1 cells, which were initially thought to drive autoimmune diseases, is now debated. The requirements for Th17 differentiation are more complex than for Th1 and Th2 cells, because IL-17 production in CD4^+^ T cells can be induced by different cytokine combinations. Initially, TGF-β plus IL-6 was identified in mice (105), while IL-1β, IL-6, and/or IL-23 were proposed in humans (106, 107). The *de novo* Th17 differentiation is very inefficient in humans, and therefore it was suggested that only a cocktail with all four cytokines induces significant Th17 differentiation (108). Although the role of TGF-β in human Th17 differentiation has been a subject of debate (109), it was shown in mice that TGF-β induces ROR-γt, while pro-inflammatory cytokines are required to inhibit TGF-β-induced Foxp3 expression and thus Treg generation (110). The presence of CD4^+^ T cells co-expressing Foxp3, RORC2, and/or IL-17 in humans is consistent with a role for TGF-β in human Th17 and Treg development (86, 88). An alternative explanation for the positive role of TGF-β in Th17 differentiation is that TGF-β indirectly favors Th17 cell differentiation by inhibiting Th1 cell development (111). Indeed, in the absence of TGF-β1 (106, 107, 112), or in the presence of TGF-β3 in mice (113), pathogenic Th17 cells that co-produce IL-17 and IFN-γ are generated. These Th1/17 cells co-express RORC2 and T-bet, are enriched in autoimmune patients and are specific for both Th1 and Th17-inducing pathogens (114, 115).

*In vitro* stability experiments and fate reporter mice suggested that Th17 cells are partially unstable and can switch completely from IL-17 producing Th17 to IFN-γ producing Th1 cells in chronic immune responses (92, 116). IL-12 can induce this Th17-to-Th1 switch (117), and CD161 was proposed as a marker that distinguishes these ex-Th17 cells from conventional Th1 cells in humans (118). However, *ex vivo* isolated human Th17 cells exhibited stable epigenetic marks at cytokine and transcription factor loci (119), suggesting that *in vivo* generated human Th17 cells are not necessarily unstable. Finally, also a very rare population of human T cells that co-produces IL-17 and IL-4 was identified (120). These Th2/17 cells were proposed to be highly pro-inflammatory in allergic asthma, but their role in immune responses against pathogens remains to be understood.

Th17 cells are highly heterogeneous and produce several effector cytokines besides IL-17. IL-22, a cytokine that promotes epithelial proliferation and barrier function (121), is produced by some Th17 cells (122, 123), and IL-22 and IL-17 co-operate to control gram-negative bacteria in the lung (124). However, a subset of human skin-homing IL-22 producing cells was identified that were distinct from Th17 cells (125, 126). Indeed, in contrast to IL-17, IL-22 is inhibited by TGF-β (127) and thus how Th17 cells acquire IL-22 producing capacities and if they can even switch from IL-17 to IL-22 production is unclear. Some Th17 and Th22 cells also produce IL-26, a pro-inflammatory cytokine that is not expressed in mice (128) and that also acts selectively on non-hematopoietic cells. A particular relevant cytokine in the pathogenesis of experimental autoimmunity is GM-CSF, which is induced by IL-1β, IL-23, and ROR-γt in mice (102, 129). Conversely, GM-CSF is inhibited by IL-1β and IL-23 in humans, and is produced by both Th1 and Th17 cells (130, 131).

Th17 cells also produce high levels of IL-21. IL-6 induces IL-21 in naive T cells upon priming (132), and IL-21 can induce its own expression (133) and promotes Th17 differentiation in an autocrine manner (131, 134–136). Importantly however, IL-21 inhibits GM-CSF and IFN-γ production and promotes instead IL-10 secretion in developing Th17 cells. Consequently, IL-21 promotes the generation of conventional (137) or regulatory Th17 cells (138), but inhibits the generation of pathogenic Th1/17 cells (131). Finally, a subset of skin-homing T cells produces IL-9 and responds to *C. albicans* (139). Some of these cells co-produce IL-9 and IL-17 (60), while others appear to represent Th9 cells. IL-9 production seems however to be transient, suggesting that these skin-homing Th9 cells are largely unstable (139).

**Figure 2:**
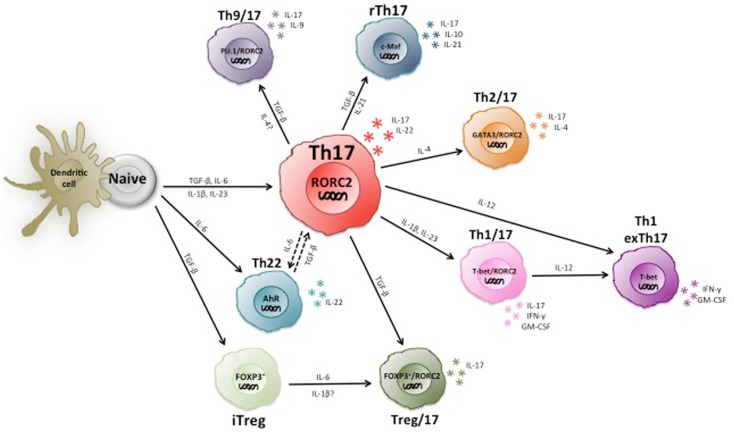
**Heterogeneity and plasticity of human Th17 cells**. Th17 cells are highly heterogeneous and produce various types of other cytokines in addition to IL-17, including the Th1 and Th2 marker cytokines IFN-γ and IL-4. Some IL-17 producing T cells express Foxp3 and/or IL-10 and are suppressive. Moreover, Th17 cells are partially unstable and can become Th1 cells upon chronic inflammation.

In summary, the current knowledge indicates that human Th17 cells are highly heterogeneous and partially unstable (Figure 2), and much remains to be learned on the role of different Th17 subsets in immune-mediated diseases.

## Regulation of Human T Cell Plasticity in Tissues: The New Frontier

The complex regulation of T helper subsets by cytokines raises the questions where T cells are re-educated and also why this might be important to successfully resist pathogens, since this was a major evolutionary pressure that shaped the human immune system. It was soon realized that stable T cell differentiation often requires repetitive *in vitro* TCR stimulation in the appropriate cytokine condition, suggesting that immature T cells might be more plastic than more differentiated ones (12, 140). *In vivo* primed T cells that are at an intermediate stage of differentiation are central memory T cells (T_CM_), which similar to naïve T cells have maintained the capacity to home to lymph nodes, produce only low levels of effector cytokines, but produce high levels of IL-2 and IL-21 (131), and expand rapidly to generate secondary waves of effector cells (8). Conversely, effector memory T cells (T_EM_) are more differentiated cells since they produce high levels of effector cytokines and home preferentially to inflamed non-lymphoid tissues (8). Consistent with the view that plasticity is progressively reduced upon T cell differentiation, pre-committed Th1_CM_ cells are more plastic than fully differentiated Th1_EM_ cells, since Th1_CM_ cells generate a substantial population of *bona fide* Th2 cells upon re-stimulation with IL-4, while Th1_EM_ cells do not revert to Th2 cells, but some acquire IL-4 in addition to IFN-γ producing capacities (24). This plasticity requires TCR stimulation, since antigen-independent proliferation with homeostatic cytokines resulted exclusively in the generation of Th1 effector cells (24). Based on these findings it can be speculated that pre-committed T_CM_ cells that cross-react with a different pathogen can be still partially re-educated to a different lineage in lymph nodes, while T_EM_ cells do not easily switch cytokine production, but rather become polyfunctional (Figure 1). Another example of functional plasticity in lymphoid organs is the generation of follicular Foxp3^+^BCL-6^+^ Tregs, which are specialized Tregs that control B cell responses (79, 80). Also Tregs in non-lymphoid tissues acquire tissue-specific properties that are important for their functions (76). T_EM_ helper cells that are activated by antigen in non-lymphoid tissues can up-regulate CCR7 (141) and home to inflamed lymph nodes (142) where they can influence the secondary immune response and are exposed to a different cytokine milieu. Conversely, tissue-resident memory (T_RM_) cells have lost sphingosine-1 phosphate receptors and thus also the capacity to re-circulate through the blood to secondary lymphoid organs (143). T_RM_ belong predominantly to the CD8 compartment, but influenza virus-specific CD4^+^ T_RM_ can be identified in the lung of humans and mice (144). If tissue-resident CD4^+^ T cells are terminally differentiated effector cells or still possess the plasticity to acquire additional cytokine producing capacities remains to be established (145).

A central organ for the generation of different subsets of Th17 cells is the intestine (146). Thus, upon self-limiting colitis induced by anti-CD3 injections in mice predominantly IL-10 producing Th17 cells with regulatory functions are induced (138). Conversely, under conditions that induce IL-23 in the intestine pathogenic IFN-γ and GM-CSF producing Th17 cells are generated that induce colitis (147, 148). IFN-γ and IL-17 co-producing Th1/17 cells have also been observed in patients with IBD (92), but very little is known about the regulation of Th17 responses in the human intestine. Th1/17 cells that produce IL-17, IFN-γ, and GM-CSF also drive central nervous system (CNS) inflammation in EAE, a standard mouse model of multiple sclerosis (MS) (149). The CNS is separated from pro-inflammatory T cells by the blood–brain barrier (150), but spontaneous JC Virus re-activations and progressive multifocal leukoencephalopathy in MS patients treated with anti-VLA-4 antibodies, which block lymphocyte extravasation to the CNS, suggest nevertheless a constant immune surveillance by T cells (151). How the microenvironment of the CNS influences the properties of CD4^+^ T cells is the focus of intensive research in mice, but is largely unknown in humans given the difficulties to analyze T cells in the human CNS.

Thus, accumulating evidence underlines the role of the tissue microenvironment in T cell plasticity, and the identification of tissue-specific factors that control T cell functions is likely to have a major impact on translational medicine.

## Conclusion and Perspective

The original concept of two terminally differentiated subsets of Th1 and Th2 cells has been substituted by the view that many different T cell subsets with specific cytokine profiles are required to protect us from the different pathogenic insults that were are continuously exposed to. These various T cell subsets possess different degrees of plasticity to acquire new characteristics and functions in secondary or chronic immune responses. In particular, while the stability of Tregs is debated, it is widely accepted that Th17 cells are largely unstable, although exceptions might exist. In addition, human Th17 cells are highly heterogeneous, but the functions of all these different types of Th17 effector cells in protective immune responses and their roles in autoimmune diseases remain to be understood. Another important but poorly understood aspect of T cell plasticity is how different tissue microenvironments impact on human T cell differentiation and stability. The definition of the relative plasticities or stabilities of human T cell subsets in different tissues is highly relevant for future therapeutic interventions in so different immune-related pathologies as chronic viral infections, cancer, and autoimmune diseases.

## Conflict of Interest Statement

The authors declare that the research was conducted in the absence of any commercial or financial relationships that could be construed as a potential conflict of interest.
